# Endocrine–circadian interactions in birds: implications when nights are no longer dark

**DOI:** 10.1098/rstb.2022.0514

**Published:** 2024-03-25

**Authors:** Barbara Helm, Timothy Greives, Michal Zeman

**Affiliations:** ^1^ Swiss Ornithological Institute, Bird Migration Unit, Seerose 1, 6204 Sempach, Switzerland; ^2^ Department of Biological Sciences, North Dakota State University, Fargo, ND 58102, USA; ^3^ Department of Animal Physiology and Ethology, Faculty of Natural Sciences, Comenius University, Bratislava SK 84215, Slovakia

**Keywords:** melatonin, clock, light pollution, ALAN, annual timing, development

## Abstract

Biological clocks are evolved time-keeping systems by which organisms rhythmically coordinate physiology within the body, and align it with rhythms in their environment. Clocks are highly sensitive to light and are at the interface of several major endocrine pathways. Worryingly, exposure to artificial-light-at-night (ALAN) is rapidly increasing in ever more extensive parts of the world, with likely impact on wild organisms mediated by endocrine–circadian pathways. In this overview, we first give a broad-brush introduction to biological rhythms. Then, we outline interactions between the avian clock, endocrine pathways, and environmental and internal modifiers. The main focus of this review is on the circadian hormone, melatonin. We summarize information from avian field and laboratory studies on melatonin and its relationships with behaviour and physiology, including often neglected developmental aspects. When exposed to ALAN, birds are highly vulnerable to disruption of behavioural rhythms and of physiological systems under rhythmic control. Several studies suggest that melatonin is likely a key mediator for a broad range of effects. We encourage further observational and experimental studies of ALAN impact on melatonin, across the full functional range of this versatile signalling molecule, as well as on other candidate compounds at the endocrine–circadian interface.

This article is part of the theme issue ‘Endocrine responses to environmental variation: conceptual approaches and recent developments’.

## 1. Introduction

**Figure 1.**
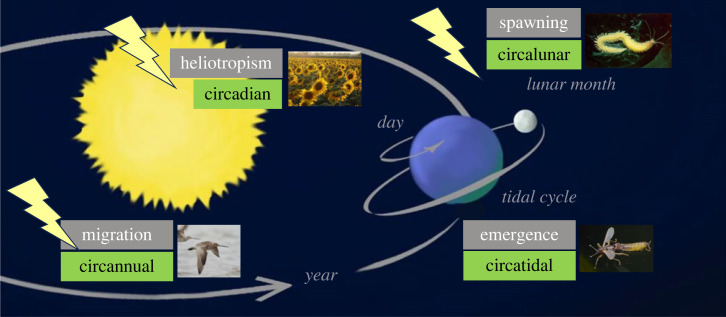
Biological rhythms, their planetary foundations, and their sensitivity to light. Organisms on Earth experience highly predictable planetary movements on time scales of a year, a day, and of variants of lunar and tidal rhythms. These rhythms shape the light environment and thereby a myriad of biological processes, exemplified by the inlays. Organisms' clocks are highly sensitive to photic cycles, but artificial light at night (ALAN) interferes with perception of natural light, indicated by bright yellow flashes. Image: Edda Starck, and WIKI commons. (Online version in colour.)

Life on our home planet is shaped by predictable rhythmicity on many time scales (figure 1). These include Earth's elliptical orbit around the Sun on a tilted axis, generating annual cycles, Earth's rotation around its axis, generating diel cycles, and Moon's orbit around Earth, generating lunar and tidal cycles. The primary terrestrial manifestation of annual, diel and lunar rhythmicity on Earth are cyclical changes in the availability, direction and composition of light (figure 1). Indirectly, this causes many environmental factors to fluctuate, such as ambient temperature, water availability and biotic context, which in turn are essential for the life of organisms. It is thus little wonder that a myriad of biological processes align with planetary periodicities (figure 1), ranging from molecular to ecosystem levels. Perhaps more surprisingly, such an alignment is not simply a response to environmental fluctuations, but is based on an internal representation of time through biological clocks.

Biological clocks evolved an estimated 2–3 billion years ago [1,2]. It is thought that these clocks aided cyanobacteria in acquiring resources during light availability, while conducting sensitive processes, such as DNA-replication, during dark hours without risk of UV damage. Benefits of clocks are thus principally twofold: ‘external’ with respect to the environment (i.e. for alignment and anticipation), and ‘internal’ by maintaining temporal order (i.e. for physiological coordination and compartmentalization) [1]. Circadian clocks, which organize diel timing, are found in most organisms, whereas circannual, circalunar and circatidal clocks have been described in fewer species, albeit also across the tree of life [3–6].

Underlying principles of clocks are similar across time scales: organisms keep track of time through a combination of endogenous rhythm generation and use of specific environmental entraining cues (i.e. *Zeitgebers*). *Zeitgebers* are cues from the environment that shift an organism's internal clock, rather than having just a momentary effect on processes like sleep, feeding or locomotor activity. Thus, organisms use as *Zeitgebers* highly reliable cues with strong predictive power, which predominantly relate to properties of light (e.g. sunrise, sunset, day length). *Zeitgebers* can also include, e.g. clearly timed patterns in ambient temperature, food availability, social factors, predation risk, and, for tidal cycles, gravitational forces [4,7].

The internal component of time-keeping can be demonstrated when organisms are isolated from all rhythmic environmental information, but continue to display rhythmic processes. However, without environmental input, period lengths commonly deviate from planetary rhythms, and rhythmic processes can drift (i.e. free-run) and disintegrate [7]. Thus, proper interfacing of internal clocks with the environment is crucial for biological time-keeping [1]. This interfacing is well-organized within time-keeping systems. Clocks are highly sensitive to *Zeitgebers* and respond to them depending on the phase of their rhythm [1,7]. Thus, the same environmental cue can have opposite effects on an organism's behaviour or physiology. For example, light exposure prior to expected sunrise advances the circadian clock whereas light exposure after expected sunset delays it. Similarly, on an annual time scale, organisms also respond to *Zeitgebers* depending on the phase of their rhythm. For example, in birds, exposure to long days in spring advances the circannual clock whereas exposure to long days in autumn delays it [3,8].

Hormones are at the main interface between clocks and environment, and play key roles for temporal coordination. Their contributions range from transduction and amplification of *Zeitgeber* cues, through pacing of clock components, to dissemination of clock information and modulation of physiological responsiveness throughout the body [9–12]. Clocks are thus intimately coupled to several major endocrine pathways on diel and annual time scales.

As an interface between clocks and the environment, hormones also mediate many effects of artificial light at night (ALAN) on organisms [12–15]. The focus of this review is on the major circadian hormone, melatonin, which is highly sensitive to light [16]. Melatonin is of central importance because it conveys photic information, and thereby also ALAN effects, within the organism [17]. However, other hormones involved in temporal coordination can also be affected by light pollution (see below). For example, in wild species, changes in rhythmic patterns have been demonstrated for corticosteroids and their receptors, and for reproductive hormones and their receptors [12,15,18,19]. Evidence for ALAN-induced endocrine disruption for a wider range of hormones derives mostly from laboratory rodents [20]. For wild species, studies commonly reported changes in overall hormone levels in response to ALAN, in particular for corticosteroids [12].

Negative impacts of ALAN have long been identified in biomedical, veterinarian and sociological research that documented a broad range of effects of clock disruption [15,21,22]. Likewise, ecological effects of ALAN are now also well-documented [13,23,24]. Worryingly, ALAN, and thereby light pollution defined as biologically active artificial light in the environment, is rapidly increasing in extent and intensity [25]. Affecting biological rhythms on a global level (figure 1), ALAN is now considered a major ecological threat [24,26]. Changes in diel and annual timing as a consequence of ALAN are widespread across taxa. A recent meta-analysis has summarized published evidence for impact of ALAN on physiology, activity patterns, life history and communities across the tree of life [24]. One of the unexpected conclusions was that ALAN overall impacted nocturnal and diurnal endotherms to similar extent. While activity and life history of birds and mammals were more affected for nocturnal species, as predicted by the authors, effects on physiology were greater for diurnal species [24].

Effects on timing are particularly well explored in birds. In this predominantly diurnal taxon, diel activity generally starts earlier in the day during ALAN exposure in wild and captive settings [24]. Similarly, spring activities, such as breeding and return migration, are commonly advanced, whereas autumnal activities, such as outward migration, may be delayed by ALAN [18,27]. Several overview articles have documented major effects on avian physiology, including on melatonin and on other endocrine pathways that are closely linked to time-keeping [12–14,21,24]. Here, we seek to exemplify the endocrine-clock interface from environmental and physiological perspectives for birds, and then summarize evidence for implications of ALAN exposure. For more comprehensive and detailed coverage of ALAN effects, we refer readers to excellent published reviews [12–15,21,23,24,28].

## 2. Endocrine–clock interactions in avian physiology

**Figure 2.**
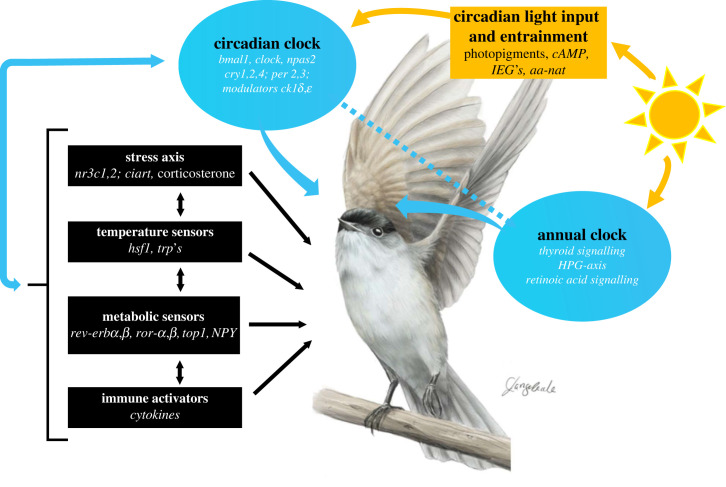
Pathways involved in avian time-keeping. Timing results from multiple bi-directional interactions between endogenous circadian and annual clocks, light input, and environmental and internal modifiers like stress, nutrients, ambient temperature and immune state. Clock components are shown in blue circles, light input pathways in a yellow box and additional pathways in black boxes. The schematic shows only exemplary compounds of pathways, and only some interactions between pathways. *aa-nat*, aralkylamine N-acetyltransferase; *ciart*, Circadian Associated Repressor of Transcription; *ck1δ,ε*, casein kinase I isoform delta, epsilon; *clock*, Circadian Locomoter Output Cycles Protein Kaput; HPG, hypothalamic-pituitary-gonadal axis; *hsf*, Heat Shock Transcription Factor; IEG, immediate early genes; *npas*, Neuronal PAS Domain Protein 2; NPY, Neuropeptide Y; *nr3c*, nuclear receptor subfamily 3, group C; *reverbα,β*, nuclear receptor subfamily 1, group D member 1,2; *rorα,β*, RAR Related Orphan Receptor isoform A,B; *top,* DNA topoisomerase I; *trp*, transient receptor potential; and see text. Illustration: Blackcap (*Sylvia atricapilla*) by Corinna Langebrake.

In vertebrates, circadian rhythms are primarily generated through transcription–translation feed-back loops of clock genes within cells [10,11]. Among avian core clock genes, unlike in mammals, *per1* (Period Circadian Regulator 1) is not expressed in birds [11], and cryptochromes (encoded by *cry*- genes) might have photoreceptive capacities [29]. The core clock loop is connected with auxiliary feed-back loops (figure 2). One auxiliary loop links metabolism with the central clock through metabolic sensors that interact with a core clock gene, *bmal1* (Basic Helix-Loop-Helix ARNT Like 1; aka *arntl*). Another loop links to stress systems also via interactions with core clock genes that modulate corticoid receptor function over the day [30]. The core loop and some auxiliary loops are also tightly integrated with the immune system in a bi-directional way [31–33]. Light information is integrated in the molecular clock through action of cAMP (cyclic adenosine monophosphate) response elements on core clock genes and immediate early genes [34,35]. The core clock loop is linked to melatonin by activating the expression of synthesizing enzymes [36], and conversely through melatonin feed-back on *bmal1* [37].

Similarly, in several other pathways, endocrine–circadian links on molecular levels are complemented by rhythmic patterns of the hormone itself, and by two-way interactions of pathway compounds with biological rhythms. For example, corticosterone represents the major output of the hypothalamic–pituitary adrenal (i.e. HPA) axis and exhibits a distinct circadian rhythm with the highest levels before activity onset [38]. This general pattern is widespread among birds, with some exceptions, e.g. at high latitudes, but it undergoes seasonal modulation [39–41]. Sex steroids, such as circulating testosterone and estradiol concentrations, also exhibit distinct circadian rhythms [42,43], and in turn, can exert powerful effects on the circadian system [44,45]. Some of the pathways that interact with biological rhythms are summarized in figure 2.

**Figure 3.**
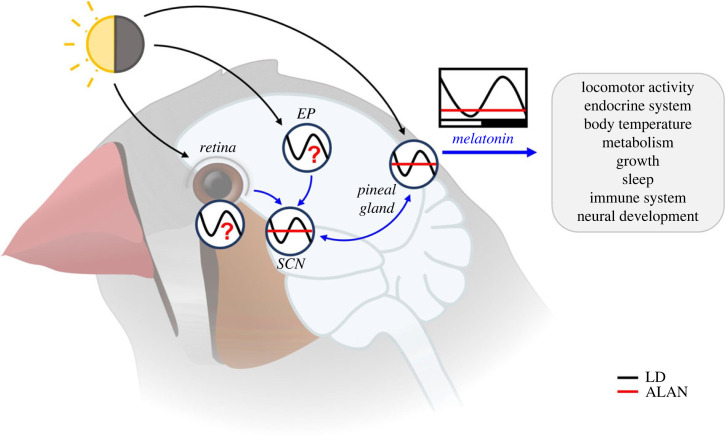
The central circadian system of birds. It consists of three self-sustained oscillators, the SCN, the pineal gland and the eyes. The SCN receives light information from the encephalic photoreceptors (EP), while the pineal gland and eyes have their own photoreceptors. They communicate via melatonin and can all be differentially affected by ALAN. Suppressed melatonin levels may indicate weakened coupling between oscillators and reduced circadian control of behavioural and physiological rhythms. Black lines show rhythmicity under natural light-dark cycles, red lines indicate rhythmicity suppressed by ALAN. Image shows a zebra finch (*Taeniopygia guttata*).

Within the circadian system, the many cell-based oscillators are usually synchronized within an organism [19]. The circadian system has pacemaker functions in specific tissues, with main control centres in the brain, and peripheral clocks in all organs [46]. In mammals, the main control centre is located in the suprachiasmatic nuclei (SCN) of the hypothalamus, which relays time information across the body, including to the pineal gland, the main production site of melatonin. In birds, the central part of the circadian system is more complex (figure 3). In addition to the SCN, the avian pineal gland and retinas can also generate self-sustained circadian oscillations under constant conditions [11,35,47,48]. Moreover, birds possess extraocular encephalic photoreceptors and can therefore be synchronized to light:dark (LD) cycles in the absence of retinal photoreceptors after enucleation [49–51].

The circadian system is also important for annual timing through photoperiodism, as shown by multiple behavioural experiments [10]. On a circannual time scale, rhythm generation is poorly understood [52]. Photoperiodic information informs annual cycles through at least two pathways, one involving thyroid signalling, and another implicating retinoic acid signalling [53–55]. These pathways also integrate information on further organismal states, for example on nutrition [56] (figure 2). Photoperiodic input pathways of birds use deep-brain photoreceptors, in contrast to mammals, where melatonin plays a key signalling role [13,57]. Mammalian and avian photoperiodism converges in thyroid pathways and in further action on the reproductive (i.e. hypothalamic–pituitary–gonadal (HPG)) axis [10,57].

## 3. Melatonin: the circadian hormone of birds

### (a) Main features of melatonin

Melatonin, an evolutionary ancient molecule, is synthetized in vertebrates, invertebrates, plants and some bacteria, including cyanobacteria [2,17,58]. Melatonin concentrations are characterized by distinct daily rhythmicity with high concentrations at night and low concentrations during the day. The main site of melatonin production in birds is the pineal gland, although in some species, eyes can also be major contributors [35,50]. Pineal glands cultured *in vitro* and isolated pinealocytes in culture express circadian patterns of melatonin production and these rhythms persist in continuous darkness, indicating their endogenous nature [17,47].

Melatonin is a derivative of the amino acid tryptophan and is formed by a four-step biosynthesis. Tryptophan is hydroxylated to form 5-hydroxytryptophan by tryptophan hydroxylase, which is the rate-limiting step for serotonin production. In the pineal gland, serotonin is converted by the enzyme aralkylamine *N*-acetyltransferase (AA-NAT) to N-acetylserotonin, from which hydroxyindole-*O*-methyltransferase generates melatonin [59].

The characteristic diel pattern of melatonin is determined by circadian activity of AA-NAT, which is controlled by the sympathetic system via adrenergic receptors on pinealocytes. In birds, norepinephrine inhibits melatonin biosynthesis during the day. Moreover, in the chicken, norepinephrine increases amplitude and decreases damping of the melatonin rhythm in constant darkness but does not phase-shift the pineal circadian clock [60]. Rhythmic melatonin can also be found in other avian tissues, such as gut [61]. It is possible that melatonin measured in the gastrointestinal tract is produced at least partially by microbiota, and has been recently claimed to have protective effects on the host [62,63].

### (b) Diversity of melatonin mechanisms and patterns

Although melatonin is ancient and widely involved in rhythmic organization, its particular role in a given species, and some main functions and underlying mechanisms, appear to be quite variable. For example, melatonin and its main secretory gland, the pineal, seem to be more important for circadian organization in birds than in mammals, because pinealectomy in mammals does not disturb overt circadian rhythms of locomotor activity as it does in birds [35]. Furthermore, sympathetic control of melatonin is inverted between birds and mammals [60], but the final diel rhythm has the same characteristic pattern in both taxa.

**Figure 4.**
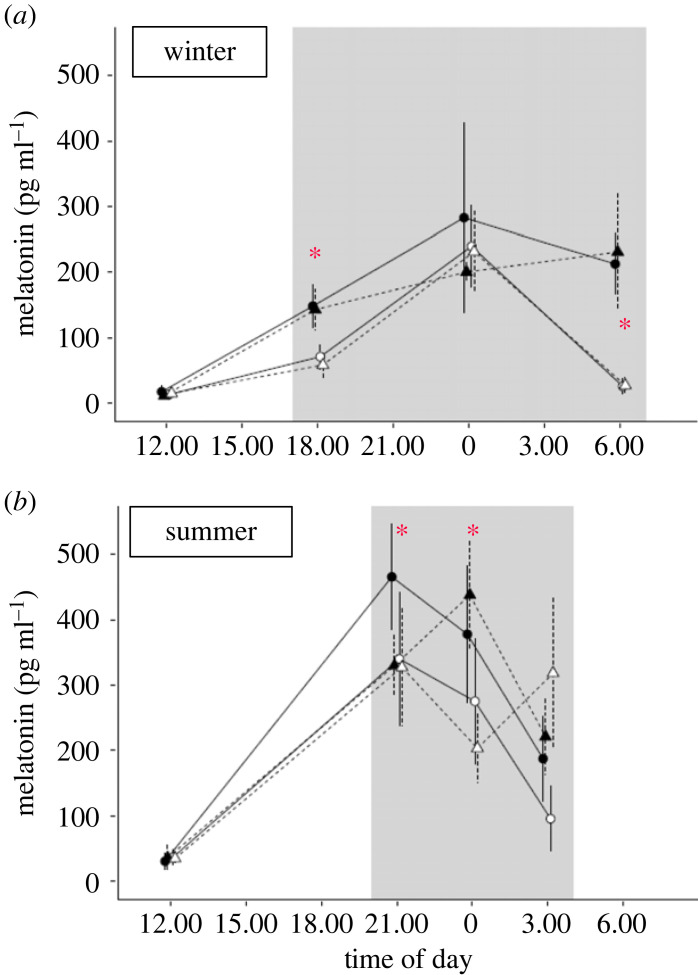
Variation in diel melatonin concentration of European blackbirds (*Turdus merula*) in winter (*a*) and summer (*b*), and responses to ALAN. The experiment was carried out under simulated natural light cycles either with dark nights (black symbols) or ALAN of 0.3 lux (white symbols). Blackbirds derived either from a city (triangles) or a forest (circles). Melatonin (means ± s.e.m.) was measured each season at the start and end of the respective dark phase (grey shading), and during mid-night and mid-day in both seasons. Asterisks indicate significant effects [66].

Birds display great diversity in mechanisms and melatonin profiles, even among closely related taxa, presumably based on both phenotypic plasticity and evolutionary adjustment [64,65]. Examples of plasticity include seasonal modulation of melatonin profiles, e.g. in response to photoperiod [47,66] (figure 4), or via circannual changes in the circadian system, for example during migration [67]. Modulation also occurs in response to moonlight [68,69] and ALAN (figures 2, 3 and 4).

Examples of evolved differences are species-dependent consequences of pinealectomy and melatonin administration, possibly due to differences in the relative importance of the pineal gland and eyes in the central circadian control [70]. High-amplitude melatonin rhythms are generally found in species with clear diurnal behaviour, such as many songbirds (Passeriformes) and fowl (Galliformes). By contrast, some species whose behaviour is flexible, for example involving tidal cycles, have low-amplitude or undetectable melatonin rhythms [69]. Similarly, nocturnal birds, such as owls, typically have low-amplitude melatonin cycles, despite strong circadian rhythms [71].

Birds inhabiting polar regions exposed to continuous light often retain rhythmic melatonin profiles, but with low amplitude [41]. A recent example illustrates adjustments within the circadian system that allow sustained rhythmicity even at high latitudes. Lapland longspurs (*Calcarius lapponicus*) maintained low-amplitude melatonin cycles while breeding under continuous light at 71° N during the polar summer [72]. When these birds were exposed to constant light or darkness in captivity, melatonin amplitudes were greatly depressed, but rhythmic expression of a clock gene remained undamped in several target tissues [72]. These data indicate that melatonin cycles can be stabilized [35] under bright natural light, and that even if melatonin is suppressed, rhythmicity can persist elsewhere in the circadian system.

To date, there is no unifying theory that could explain the wide diversity of melatonin rhythmicity. However, it has been pointed out that low-amplitude rhythmicity within the circadian system facilitates entrainment to subtle or shifting *Zeitgebers*, for example during migration across longitudes, under polar light conditions, and in environments that are shaped by tidal rhythmicity [73].

### (c) Main functions of melatonin

Because of its rhythmic secretion and light sensitivity, melatonin primarily functions as a biological signal of the night (figure 3). Thereby, melatonin can be considered as the internal timing cue (*Zeitgeber*) for other organs. Rhythmic melatonin conveys information on night length (i.e. on photoperiod), encoded by duration and intensity of its nocturnal peak [47]. It also conveys information on the phase of the circadian system. For example, during short summer nights, melatonin of songbirds peaks early in the night and can decline before midnight (figure 4; [64,66]). Melatonin is involved in control of many processes, such as locomotor activity, sleep promotion, body temperature, metabolism and growth (figure 3) [17,74–76]. Although many of melatonin's actions occur in combination, comparative studies in birds suggest that they can be disentangled and may therefore entail separate mechanisms [77]. Melatonin may partly act via modulation of neurosteroids, at least in juvenile birds (see below). It also contributes to regeneration by reducing oxidative stress and by close interaction with the immune system [17,32,77–79].

Various functions of melatonin are at the interface of diel and annual behaviours. A striking example is avian migration, a time during which many species carry out migratory flights during the night [80]. During this migratory phase, nocturnal migrants show a damped melatonin rhythm, even under constant LD-cycles. The significantly lower night-time levels during migratory phases thus appear to be part of a circannual migration programme [67,81]. In turn, bramblings (*Fringilla montifringilla*) captured during spring migration and provided drinking water with melatonin reduced migratory restlessness [82]. This suggests that melatonin counteracts nocturnal migration. More recently, Fusani and colleagues captured migrating warblers, treated them with melatonin applied topically on the skin and observed migratory behaviour overnight. Melatonin treatment reduced night-time migratory restlessness in wild songbirds treated during autumn migration but not during spring migration [83,84].

The encoding of night length through melatonin generates seasonal information, which in temperate-zone mammals can organize or modulate a host of seasonally changing processes (e.g. reproductive condition, immune function, aggressive behaviour, metabolism [17,21,85]). By contrast, for birds, melatonin is usually not required for induction of reproductive condition or the maintenance of annual cycles. For example, birds whose melatonin rhythm was abolished by pinealectomy still showed annual gonadal and moult cycles [86,87]. However, pinealectomy modulated several seasonal functions, possibly through damped or altered circadian rhythms [86]. Injections of melatonin or antibodies against melatonin in quail modulated testicular growth [88,89]. Furthermore, a hormone that regulates the termination of avian reproductive condition, gonadotropin-inhibitory hormone (GnIH, the avian analogue of RFRP-3), is responsive to melatonin manipulation, both centrally and in the gonads [90,91]. GnIH may thus be one potential mechanism whereby melatonin could influence reproductive timing decisions. Melatonin also affected further seasonal functions, such as neuroplasticity. In European starlings (*Sturnus vulgaris*), melatonin treatment altered the size of the HVC (formerly High Vocal Centre). This brain region influences bird song, a behaviour that is critical for reproduction [92].

While these studies were predominantly carried out in male birds, melatonin could have further effects in females. Full maturation of follicles in oviparous females requires additional tissues beyond the gonads (e.g. liver). Intriguingly, avian livers express melatonin receptors [93], suggesting that melatonin may be capable of influencing yolk-precursor production.

Very few studies have investigated a role of melatonin in free-living birds. One exception to this were studies on great tits (*Parus major*), which observed that melatonin manipulation led to a delay in the onset of both clutch initiation and diel activity [94,95]. The melatonin-induced delay in clutch initiation differed between two study years, being greater in the colder and wetter year. The data suggest that melatonin indeed influences physiological systems regulating timing of clutch initiation, and may influence how these systems balance inhibitory (e.g. signals of short days) and stimulatory (e.g. temperature) cues. As the climate is changing, spring weather is becoming more unpredictable, and birds that breed in response to cues of a ‘false-spring’ suffer fitness costs [96–98]. Effects of ALAN on melatonin synthesis and release (see below) may impact the sensitivity of females to a diverse range of cues, with implications for reproductive success in a changing world. More work is needed to clarify a potential role for this endocrine signal of ‘season’ to calibrate physiological systems regulating female laying decisions.

In males, the melatonin-induced delay in diel behaviour was fitness-relevant, as late-rising males lost paternity in the clutches of their female mates [95].

### (d) Ontogeny of melatonin in birds

Rhythmic melatonin biosynthesis in birds starts already during embryogenesis, and thus earlier than in mammals [99]. In precocial chicken, melatonin biosynthesis in the pineal gland starts in the second third of embryonic life [100], with similar patterns reported in geese [101]. Rhythmicity of AA-NAT has been detected by embryonic day 16, and its amplitude increased progressively until hatching [102]. At early embryonic stages, the melatonin rhythm can be directly controlled by environmental light cycles, but is under circadian control in 18-day-old embryos because it persists for 2 days in constant darkness [103]. In clock genes, rhythmic oscillations also start to develop during embryonic life in the chicken pineal gland and SCN [104]. Previous experience of the embryo with a LD cycle is required for detectable rhythmicity [105], most likely for synchronization of single oscillators in the studied structures. Therefore, both circadian clockworks and rhythmic melatonin biosynthesis start to function during embryonic life in the pineal gland. Whereas this may be expected in precocial chicken, importantly, a distinct melatonin rhythm immediately after hatching was also recorded in altricial starlings [106] and zebra finches [107].

The early development of melatonin rhythmicity in birds, compared to mammals, is most likely an adaptation to their extrauterine development and the absence of direct maternal signals [108]. It is possible that the main functions of melatonin in adult birds also apply to juvenile and perinatal stages. Additionally, early melatonin cycles could be important for ontogeny and for shaping other diel rhythms within offspring. For example, exogenous melatonin decreased heart rate of avian embryos [109]. Some of melatonin's developmental actions might be via modulated neurosteroids. During the early posthatch period, neurosteroids are synthesized predominantly in the pineal gland [110]. For example, biosynthesis of 7α-hydroxypregnenolone is modulated by melatonin, and its intracerebro-ventricular injection stimulated spontaneous locomotor activity in a dose-dependent manner in chicks [111]. We speculate that melatonin rhythms might epigenetically modulate the ontogeny of other rhythms or the whole circadian organization. Research on cell lines indicates potential epigenetic effects of melatonin by DNA methylation and histone protein remodelling [112]. On an organismic level, rhythmic brooding of quail chicks had epigenetic effects on circadian organization and long-term effects on behaviour and physiology [113].

External reasons for early melatonin cycles could be synchronization with parents and efficient coping with periodic environmental conditions. A possible ecological relevance is illustrated by the magnitude of environmental fluctuations experienced *in ovo*. In nest-box-breeding starlings, recordings from dummy eggs showed substantial diel increases in light intensity, concurrent with drastic declines in temperature, during daytime when parents left the nest [114]. Such possibly synchronizing effects of ambient temperature on embryos could be dampened in an increasingly warming world. Effects of temperature on embryonic growth have been experimentally documented in free-living passerine birds, but synchronizing aspects of so-called ‘ambient incubation’ were not investigated [115]. Furthermore, temperature-mediated changes of melatonin production could play a role in the adaptation of embryos to low temperature, given melatonin's effects on body temperature and energy metabolism in adults, but experimental data in embryos are needed.

**Figure 5.**
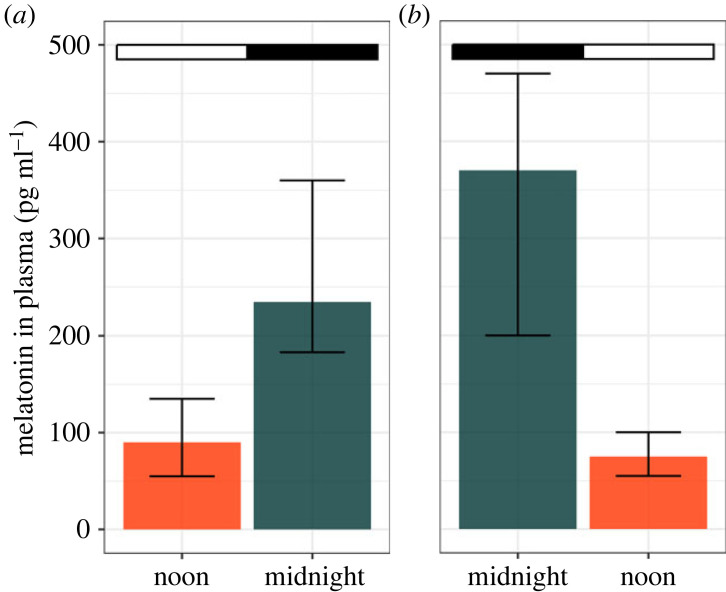
Hatchling melatonin synchronizes to low-amplitude light–dark cycles. Starling eggs were exposed to alternating dim light (10 lx) and darkness in incubators for 4–5 days before hatching. One group (*a*) was exposed to natural light-cycles, the other (*b*) to inverted cycles. Melatonin (medians ± interquartile range) in plasma aligned with these cycles; based on data from Gwinner *et al.* [114].

In the above nest-box study on starlings, the recorded light intensity fluctuations were applied to eggs in incubators to test effects on melatonin (figure 5). Hatchlings collected *ca* 10 h post-hatching had melatonin concentrations that aligned with these dim-light–dark cycles. Synchronization was corroborated by setting light cycles in one incubator synchronous to local time, whereas in a second incubator dim-light cycles were phase shifted by 12 h. In both incubators, hatchlings had significantly higher pineal and plasma melatonin concentrations during the respective dark phases than during the light phases (figure 5) [114]. Thus, ambient light clearly exerts strong perinatal effects, but how light and temperature interactively affect clock ontogeny is unknown for songbirds. To our knowledge, this research line has not been continued in wild birds. However, research on chicken has detailed wavelength-specific effects of light on embryonic melatonin rhythms [116]. In addition, ambient temperature cycles can also entrain embryonic rhythmicity and affect melatonin levels in chicken [117]. When both, light and temperature cycles were applied, embryos responded more strongly to light, and temperature had a modulatory role [117]. Thus, similar to findings of temperature-dependent effects of melatonin on clutch initiation (see above), these data also suggest interactive effects of light and temperature on melatonin patterns, which need to be explored in a warming world.

## 4. Impacts of light pollution on birds

Changes in avian physiology were already reported soon after electric lighting spread in starlings that rested below streetlights in London [118]. More than 80 years later, evidence for light-induced changes in behaviour of free-living and captive organisms is pervasive [13,23,24]. The most consistent observation is advanced diel activity onset under ALAN [24]. Annual phenology was also modified, but the direction of change varied, possibly due to species-specific and phase-specific photoperiodic responses [18,24]. Changes in behavioural rhythms were investigated both in the laboratory and in the field. Overall, patterns in these settings were similar, but ALAN effects in the laboratory were typically much stronger. This could be due to limitations of simulating the many features of natural light in the laboratory, to the complexities of fieldwork, where conditions cannot be controlled, and to differences in state of animals in captivity and the wild [72,119]. Nonetheless, as the overall trends are similar, the respective strengths of field (realistic scenarios) and laboratory (causal inference) jointly corroborate the evidence.

Many further aspects of avian behaviour are vulnerable to light pollution. For example, nocturnally migrating birds use stars for navigation, based on stellar positions they learn during development [120]. This behaviour requires heightened visual sensitivity at night and dark skies with visible stars. In fact, ALAN effects on migratory birds are a major conservation concern. Birds may be attracted or repelled by ALAN. On close distance, many lose orientation and suffer fatal collisions with human infrastructure [121,122]. ALAN has also major ecological effects on birds, such as changes in prey availability, in predation risk and in complex ecosystem functions [24,123,124].

Among the vast physiological effects of light pollution, many affect rhythmic features or arise from circadian or seasonal disruption, as laid out in recent overview articles [12–15,21,24,28]. A slate of studies, reviewed therein, have elaborated important general features of physiological responsiveness. Briefly, these include extremely high sensitivity, whereby birds responded to ALAN below 1 lux (figure 4). Secondly, responses were dose-dependent. Thirdly, responses were wave length-dependent. Rhythmic processes of birds were typically most affected by blue light, or by white light containing high contributions from the blue spectrum [13,14,28]. These patterns were broadly generalizable across further organisms, although intensity thresholds and spectral sensitivities varied between taxa.

Physiological changes were particularly evident in hormones. Many endocrine-linked measures were affected by ALAN, but except for those on melatonin, most studies were not tailored to address diel rhythmicity, and responses were typically diverse [19,24]. Disruptive effects of ALAN on plasma glucocorticoid levels have been reported in several studies. One study with a detailed sampling routine on zebra finches showed a clear damping of corticosterone rhythms alongside melatonin rhythms under ALAN [125]. A damping of rhythms in corticosterone and its receptors was supported by several other studies, which however were measured only at one or two time points over a 24 h cycle [12,18,19]. Research on rodents advises caution for conclusions from studies with few time points which may lead to contradictory findings [22]. When in a study on rats the complete daily rhythm was assessed after whole-night exposure to 2 lux, corticosterone rhythmicity was seen not to be abolished. Rather, it was preserved, but the rhythm was phase-advanced and had a lower amplitude [20]. The shift may result in earlier awakening, interfere with sleep quality and can phase-advance physical activity. Overall levels of corticosteroids, integrated across the day through measurement of faeces or feathers, mostly indicated increased activity of the HPA axis [12].

Studies of ALAN effects on diel rhythms in avian reproductive hormones are to our knowledge missing, and this represents a serious gap in our understanding. For example, testosterone regulates various reproduction-related processes, and an absence of its daily oscillations, as shown in ALAN-exposed rats [20], may profoundly affect male reproductive function and behaviour. However, a recent study on great tit males showed that ALAN exposure, which greatly accelerated reproductive activation, also had major effects on day–night patterns of various transcripts of reproductive pathways in the testes, including of receptors for reproductive hormones [18].

On an annual time scale, ALAN effects on time patterns of reproductive hormones and other reproductive features have been shown in correlational field studies and in experimental laboratory studies [18,126,127]. Overall, these physiological effects aligned well with the observation of advanced reproductive behaviour. It is likely that most changes to reproductive cycles arose through photoperiodic pathways [21]. However, experimental manipulation of melatonin also altered lay date (see above [94]). Thus, ALAN may also alter via melatonin how female birds calibrate relevant environmental cues to time seasonal breeding.

Close links between the avian immune and circadian systems have been corroborated by studies on chicken which emphasized a regulary role for melatonin [33]. For wild birds, there is increasing evidence of immuno-suppression in direct response to ALAN, as well as increased susceptibility to diseases in the wild. Some studies that simultaneously investigated the immune system and circadian biology proposed that physiological effects were mediated by melatonin suppression or by desynchronization between host and parasite [128–130]. Additionally, modification of annual cycles through ALAN effects on photoperiodism could have also affected avian immune features or desynchronized host and parasite [131]. Presumably of benefit to health, interactions between host and gut microbiome were recently demonstrated to influence each other's diel rhythms [63]. The study on chicken proposed mediation of this interaction via melatonin, which in turn was damped by excessive lighting [63].

Endocrine–circadian responses to ALAN have been mostly studied for melatonin. For assessing rhythm-linked changes, measurements of melatonin are particularly suitable. Melatonin is not only a mediator of ALAN effects, but also a marker for the circadian system, including circadian responses to nocturnal light. Melatonin easily penetrates biological membranes and barriers to all compartments of the body, so that its concentrations can be measured in avian blood. However, recent use of commercial ELISA tests, which were not validated for avian species, may increase the risk of unrealistic values [132]. Melatonin is an indolamine and its molecular structure is identical in all taxonomic groups, so in theory its concentrations can be measured by immunoassays in samples from any species. However, immunoassays are highly dependent on the specificity of the antibody and its interference with plasma proteins, lipids, steroids, indoles, etc. As their levels vary considerably in plasma from different species, the use of commercial ELISA tests without prior validation may result in unrealistic (usually very high) values. Therefore, at least the parallelism of the immunoreactivity present in the sample against the melatonin standard curve and the recovery of a known amount of melatonin from the plasma sample should be tested to demonstrate the reliability of the measured concentrations. For further notes on measuring melatonin, see the electronic supplementary material.

Nonetheless, findings on endocrine–circadian responses to ALAN were mostly consistent for melatonin, which overall was strongly suppressed by ALAN (figures 3 and 4) [12–15,24]. Laboratory studies performed on adult birds under controlled conditions showed a dose-dependent decrease of circulating melatonin concentrations after exposure to an ecologically relevant level of light at night (0. 5–5.0 lux) in great tits [133], European blackbirds [66] and zebra finch [134]. Importantly, as expected for a circadian mechanism, the melatonin response to part-time ALAN was phase-specific, as shown in a study of migratory red-headed buntings (*Emberiza bruniceps*) [135].

During ontogeny, melatonin was measured in free-living great tit nestlings that were experimentally exposed to ALAN. Although only one blood sample was taken, around midnight, the measured reduction of melatonin concentration to 49% fits well with laboratory findings from adult birds [128]. In a laboratory experiment on chicken, neurosteroid synthesis in the pineal gland just after hatching was suppressed by ALAN [110]. Early developmental processes of melatonin are sparsely characterized, but are likely disrupted by ALAN. For example, the experimental light intensities that synchronized hatchling melatonin rhythms in starlings were within the range of ALAN experienced by free-living organisms [24,114]. In mammals, exposure of pregnant female rats to dim ALAN resulted in delayed development of melatonin rhythmicity in offspring [136] or even arrhythmicity [137]. Although research on melatonin during ontogeny is just emerging, the few existing studies thus suggest that disturbance by ALAN could have long-lasting negative effects.

## 5. Conclusion: ancient rhythms in an illuminated world

We have shown intricate and extensive relationships between the avian endocrine system and biological rhythms. In particular, melatonin, an ancient and taxonomically widespread hormone holds key roles at the endocrine–circadian interface. These roles affect all aspects of avian life, from early ontogeny through the entire life cycle. However, melatonin has species-specific roles, showing modification of its action through evolution and phenotypic plasticity.

Generally, all major aspects of the avian endocrine–circadian interface are susceptible to effects of ALAN, despite differences in strength of effect and between physiological pathways. ALAN has such extensive effects because birds have evolved exquisitely sensitive, phase-dependent responses to the light expected from predictable planetary cycles. ALAN, which deviates from these expected times and directions of light, causes physiological responses that are mis-matched to the environment, and can threaten the health, well-being and life of birds.

We thus call for reduction of light pollution and mitigation measures against impact of ALAN on wild organisms. Still, to identify pathways to mitigation, there is much more to learn through research [12,14,26]. We call for more developmental studies and for more experiments, especially in natural settings where they can help disentangle causality from correlation. In a warming world, there is also need to further investigate suggestive data on interactive effects of light and ambient temperature on melatonin-mediated processes, such as reproductive timing and clock ontogeny. Within ALAN research communities, we call for greater consideration of the tight links between physiology and biological rhythms. This includes explicit consideration of ‘time’ in experiments and sampling. Although challenging, sampling is needed over several time points across the 24 h day. It also includes measuring of aspects of behaviour and physiology beyond the most common candidates, for example linked to cognitive processes and brain development, which have been implicated in clock-mediated impact of ALAN [134]. We contend that many effects of ALAN could be symptoms of an underlying greater problem, i.e. deep circadian disruption [19], that disintegrates the rhythmic orchestration of a myriad of processes in birds and other organisms.

## Data Availability

The data are provided in electronic supplementary material [138].

## References

[1] Jabbur ML, Johnson CH. 2022 Spectres of clock evolution: past, present, and yet to come. Front. Physiol. **12**, 2526. (10.3389/fphys.2021.815847) PMC8874327 35222066

[2] Manchester LC, Coto-Montes A, Boga JA, Andersen LPH, Zhou Z, Galano A, Vriend J, Tan DX, Reiter RJ. 2015 Melatonin: an ancient molecule that makes oxygen metabolically tolerable. J. Pineal Res. **59**, 403-419. (10.1111/jpi.12267) 26272235

[3] Helm B, Lincoln GA. 2017 Circannual rhythms anticipate the earth's annual periodicity. In Biological timekeeping: clocks, rhythms and behaviour (ed. V Kumar), pp. 545-569. New Delhi, India: Springer.

[4] Rock A, Wilcockson D, Last KS. 2022 Towards an understanding of circatidal clocks. Front. Physiol. **13**, 830107. (10.3389/fphys.2022.830107) 35283768 PMC8914038

[5] Tessmar-Raible K, Raible F, Arboleda E. 2011 Another place, another timer: marine species and the rhythms of life. Bioessays **33**, 165-172. (10.1002/bies.201000096) 21254149 PMC3182551

[6] Andersen DM, Keafer BA. 1987 An endogenous annual clock in the toxic marine dinoflagellate *Gonyaulax tamarensis*. Nature **325**, 616-617. (10.1038/325616a0) 3808064

[7] Helm B, Visser ME, Schwartz W, Kronfeld-Schor N, Gerkema M, Piersma T, Bloch G. 2017 Two sides of a coin: ecological and chronobiological perspectives of timing in the wild. Phil. Trans. R. Soc. B **372**, 20160246. (10.1098/rstb.2016.0246) 28993490 PMC5647273

[8] Helm B, Schwabl I, Gwinner E. 2009 Circannual basis of geographically distinct bird schedules. J. Exp. Biol. **212**, 1259-1269. (10.1242/jeb.025411) 19376946

[9] Challet E. 2015 Keeping circadian time with hormones. Diabetes Obes. Metab. **17**(Suppl 1), 76-83. (10.1111/dom.12516) 26332971

[10] Cassone VM, Yoshimura T. 2022 Circannual cycles and photoperiodism. In Sturkie's avian physiology (7th edn) (eds CG Scanes, S Dridi), pp. 1183-1201. San Diego, CA: Academic Press.

[11] Cassone VM, Paulose JK, Harpole CE, Li Y, Whitfield-Rucker M. 2017 Avian circadian organization. In Biological timekeeping: clocks, rhythms and behaviour (ed. V Kumar), pp. 241-256. New Delhi, India: Springer.

[12] Grunst ML, Grunst AS. 2023 Endocrine effects of exposure to artificial light at night: a review and synthesis of knowledge gaps. Mol. Cell. Endocrinol. **568–569**, 111927. (10.1016/j.mce.2023.111927) 37019171

[13] Falcón J, Torriglia A, Attia D, Viénot F, Gronfier C, Behar-Cohen F, Martinsons C, Hicks D. 2020 Exposure to artificial light at night and the consequences for flora, fauna, and ecosystems. Front. Neurosci. **14**, 1183. (10.3389/fnins.2020.602796) PMC7701298 33304237

[14] Grubisic M et al. 2019 Light pollution, circadian photoreception, and melatonin in vertebrates. Sustainability **11**, 6400. (10.3390/su11226400)

[15] Zeman M, Okuliarova M, Rumanova VS. 2023 Disturbances of hormonal circadian rhythms by light pollution. Int. J. Mol. Sci. **24**, 7255. (10.3390/ijms24087255) 37108420 PMC10138516

[16] Klein DC, Weller JL. 1972 Rapid light-induced decrease in pineal serotonin N-acetyltransferase activity. Science **177**, 532-533. (10.1126/science.177.4048.532) 5050487

[17] Pandi-Perumal SR, Srinivasan V, Maestroni GJ, Cardinali DP, Poeggeler B, Hardeland R. 2006 Melatonin: nature's most versatile biological signal? FEBS J. **273**, 2813-2838. (10.1111/j.1742-4658.2006.05322.x) 16817850

[18] Dominoni DM, De Jong M, Bellingham M, O'Shaughnessy P, Van Oers K, Robinson J, Smith B, Visser ME, Helm B. 2018 Dose-response effects of light at night on the reproductive physiology of great tits (*Parus major*): integrating morphological analyses with candidate gene expression. J. Exp. Zool. A **329**, 473-487. (10.1002/jez.2214) PMC6220976 30058288

[19] Dominoni DM et al. 2022 Integrated molecular and behavioural data reveal deep circadian disruption in response to artificial light at night in male great tits (*Parus major*). Sci. Rep. **12**, 1553. (10.1038/s41598-022-05059-4) 35091579 PMC8799718

[20] Okuliarova M, Dzirbikova Z, Rumanova VS, Foppen E, Kalsbeek A, Zeman M. 2022 Disrupted circadian control of hormonal rhythms and anticipatory thirst by dim light at night. Neuroendocrinology **112**, 1116-1128. (10.1159/000524235) 35316813

[21] Liu JA, Meléndez-Fernández OH, Bumgarner JR, Nelson RJ. 2022 Effects of light pollution on photoperiod-driven seasonality. Horm. Behav. **141**, 105150. (10.1016/j.yhbeh.2022.105150) 35304351 PMC10137835

[22] Rumanova VS, Okuliarova M, Zeman M. 2020 Differential effects of constant light and dim light at night on the circadian control of metabolism and behavior. Int. J. Mol. Sci. **21**, 5478. (10.3390/ijms21155478) 32751870 PMC7432546

[23] Rich C, Longcore T. 2006 Ecological consequences of artificial night lighting. Washington, DC: Island Press.

[24] Sanders D, Frago E, Kehoe R, Patterson C, Gaston KJ. 2020 A meta-analysis of biological impacts of artificial light at night. Nat. Ecol. Evol. **5**, 74-81. (10.1038/s41559-020-01322-x) 33139919

[25] Kyba C et al. 2017 Artificially lit surface of Earth at night increasing in radiance and extent. Sci. Adv. **3**, e1701528. (10.1126/sciadv.1701528) 29181445 PMC5699900

[26] Davies TW, Smyth T. 2018 Why artificial light at night should be a focus for global change research in the 21st century. Glob. Change Biol. **24**, 872-882. (10.1111/gcb.13927) 29124824

[27] Bani Assadi S, Fraser KC. 2021 Experimental manipulation of photoperiod influences migration timing in a wild, long-distance migratory songbird. Proc. R. Soc. B **288**, 20211474. (10.1098/rspb.2021.1474) PMC8385336 34428969

[28] Yang Y, Liu Q, Wang T, Pan J. 2020 Wavelength-specific artificial light disrupts molecular clock in avian species: a power-calibrated statistical approach. Environ. Pollut. **265**, 114206. (10.1016/j.envpol.2020.114206) 32599326

[29] Xu J et al. 2021 Magnetic sensitivity of cryptochrome 4 from a migratory songbird. Nature **594**, 535-540. (10.1038/s41586-021-03618-9) 34163056

[30] Nader N, Chrousos GP, Kino T. 2010 Interactions of the circadian CLOCK system and the HPA axis. Trends Endocrinol. Metab. **21**, 277-286. (10.1016/j.tem.2009.12.011) 20106676 PMC2862789

[31] Jerigova V, Zeman M, Okuliarova M. 2022 Circadian disruption and consequences on innate immunity and inflammatory response. Int. J. Mol. Sci. **23**, 13722. (10.3390/ijms232213722) 36430199 PMC9690954

[32] Cermakian N, Lange T, Golombek D, Sarkar D, Nakao A, Shibata S, Mazzoccoli G. 2013 Crosstalk between the circadian clock circuitry and the immune system. Chronobiol. Int. **30**, 870-888. (10.3109/07420528.2013.782315) 23697902 PMC7195843

[33] Markowska M, Majewski PM, Skwarło-Sońta K. 2017 Avian biological clock – immune system relationship. Dev. Comp. Immunol. **66**, 130-138. (10.1016/j.dci.2016.05.017) 27235884

[34] Mishra I, Singh D, Kumar V. 2018 Temporal expression of *c-fos* and genes coding for neuropeptides and enzymes of amino acid and amine neurotransmitter biosynthesis in retina, pineal and hypothalamus of a migratory songbird: evidence for circadian rhythm-dependent seasonal responses. Neuroscience **371**, 309-324. (10.1016/j.neuroscience.2017.12.016) 29273324

[35] Natesan A, Geetha L, Zatz M. 2002 Rhythm and soul in the avian pineal. Cell Tissue Res. **309**, 35-45. (10.1007/s00441-002-0571-6) 12111535

[36] Ma S, Wang Z, Cao J, Dong Y, Chen Y. 2019 BMAL1 but not CLOCK is associated with monochromatic green light-induced circadian rhythm of melatonin in chick pinealocytes. Endocr. Connect. **8**, 57-68. (10.1530/EC-18-0377) 30533004 PMC6330720

[37] Beker MC et al. 2019 Interaction of melatonin and Bmal1 in the regulation of PI3K/AKT pathway components and cellular survival. Sci. Rep. **9**, 19082. (10.1038/s41598-019-55663-0) 31836786 PMC6910929

[38] Gotlieb N, Moeller J, Kriegsfeld LJ. 2018 Circadian control of neuroendocrine function: implications for health and disease. Curr. Opin. Physiol. **5**, 133-140. (10.1016/j.cophys.2018.11.001) 30957055 PMC6446932

[39] Rich EL, Romero LM. 2001 Daily and photoperiod variations of basal and stress-induced corticosterone concentrations in house sparrows (*Passer domesticus*). J. Comp. Physiol. B **171**, 543-547. (10.1007/s003600100204) 11686612

[40] Schwabl P, Bonaccorso E, Goymann W. 2016 Diurnal variation in corticosterone release among wild tropical forest birds. Front. Zool. **13**, 19. (10.1186/s12983-016-0151-3) 27152116 PMC4857432

[41] Huffeldt NP, Merkel FR, Jenni-Eiermann S, Goymann W, Helm B. 2020 Melatonin and corticosterone profiles under polar day in a seabird with sexually opposite activity-rhythms. Gen. Comp. Endocrinol. **285**, 113296. (10.1016/j.ygcen.2019.113296) 31589833

[42] Greives T, Eshleman M, Galante H, Elderbrock E, Deimel C, Hau M. 2021 Early nighttime testosterone peaks are correlated with GnRH-induced testosterone in a diurnal songbird. Gen. Comp. Endocrinol. **312**, 113861. (10.1016/j.ygcen.2021.113861) 34302846

[43] Graham JL, Needham KB, Bertucci EM, Pearson AA, Bauer CM, Greives TJ. 2019 Onset of daily activity in a female songbird is related to peak-induced estradiol levels. Integr. Comp. Biol. **59**, 1059-1067. (10.1093/icb/icz112) 31236557

[44] Elderbrock EK, Hau M, Greives TJ. 2021 Sex steroids modulate circadian behavioral rhythms in captive animals, but does this matter in the wild? Horm. Behav. **128**, 104900. (10.1016/j.yhbeh.2020.104900) 33245879

[45] Gwinner E. 1974 Testosterone induces ‘splitting’ of circadian locomotor activity rhythms in birds. Science **185**, 72-74. (10.1126/science.185.4145.72) 4836085

[46] van der Veen DR, Riede SJ, Heideman PD, Hau M, Van Der Vinne V, Hut RA. 2017 Flexible clock systems: adjusting the temporal programme. Phil. Trans. R Soc. B **372**, 1734. (10.1098/rstb.2016.0254) PMC5647281 28993498

[47] Brandstätter R, Kumar V, Abraham U, Gwinner E. 2000 Photoperiodic information acquired and stored *in vivo* is retained *in vitro* by a circadian oscillator, the avian pineal gland. Proc. Natl Acad. Sci. USA **97**, 12 324-12 328. (10.1073/pnas.200354997) 11005840 PMC17340

[48] Menaker M, Takahashi JS, Hamm H. 1980 Circadian rhythms of melatonin release from individual superfused chicken pineal glands *in vitro*. Proc. Natl Acad. Sci. USA **77**, 2319-2322. (10.1073/pnas.77.4.2319) 6929552 PMC348706

[49] Korf HW. 1994 The pineal organ as a component of the biological clock. Phylogenetic and ontogenetic considerations. Ann. N Y Acad. Sci. **719**, 13-42. (10.1111/j.1749-6632.1994.tb56818.x) 8010588

[50] Ebihara S, Uchiyama K, Oshima I. 1984 Circadian organization in the pigeon, *Columba livia*: the role of the pineal organ and the eye. J. Comp. Physiol. A **154**, 59-69. (10.1007/BF00605391)

[51] Menaker M. 1968 Extraretinal light perception in the sparrow. I. Entrainment of the biological clock. Proc. Natl Acad. Sci. USA **59**, 414-421. (10.1073/pnas.59.2.414) 5238974 PMC224688

[52] Lincoln GA, Clarke IJ, Hut RA, Hazlerigg DG. 2006 Characterizing a mammalian circannual pacemaker. Science **314**, 1941-1944. (10.1126/science.1132009) 17185605

[53] Schwartz C, Andrews MT. 2013 Circannual transitions in gene expression: lessons from seasonal adaptations. In Current topics in developmental biology (eds ER Ann, BOC Michael), pp. 247-273. New York, NY: Academic Press. 10.1016/B978-0-12-396968-2.00009-9 PMC4130376 23962845

[54] Nakao N et al. 2008 Thyrotrophin in the pars tuberalis triggers photoperiodic response. Nature **452**, 317-322. (10.1038/nature06738) 18354476

[55] Shearer KD, Goodman TH, Ross AW, Reilly L, Morgan PJ, McCaffery PJ. 2010 Photoperiodic regulation of retinoic acid signaling in the hypothalamus. J. Neurochem. **112**, 246-257. (10.1111/j.1471-4159.2009.06455.x) 19860856

[56] Helfer G, Barrett P, Morgan PJ. 2019 A unifying hypothesis for control of body weight and reproduction in seasonally breeding mammals. J. Neuroendocrinol. **31**, e12680. (10.1111/jne.12680) 30585661

[57] Nakane Y, Yoshimura T. 2019 Photoperiodic regulation of reproduction in vertebrates. Annu. Rev. Anim. Biosci. **7**, 173-194. (10.1146/annurev-animal-020518-115216) 30332291

[58] Que Z et al. 2020 Microorganisms: producers of melatonin in fermented foods and beverages. J. Agric. Food Chem. **68**, 4799-4811. (10.1021/acs.jafc.0c01082) 32248679

[59] Klein DC et al. 1997 The melatonin rhythm-generating enzyme: molecular regulation of serotonin N-acetyltransferase in the pineal gland. Recent Prog. Horm. Res. **52**, 307-357; discussion 357–8. 9238858

[60] Takahashi JS et al. 1989 The avian pineal, a vertebrate model system of the circadian oscillator: cellular regulation of circadian rhythms by light, second messengers, and macromolecular synthesis. *In Proc. 1988 Laurentian Hormone Conf. (Recent progress in hormone research, vol. 45)*, pp. 279-352. Boston, MA: Academic Press. 10.1016/b978-0-12-571145-6.50010-8 2682842

[61] Herichova I, Michal Z, Veselovský J. 1998 Effect of tryptophan administration on melatonin concentrations in the pineal gland, plasma and gastrointestinal tract of chickens. Acta Vet. Brno **67**, 89. (10.2754/avb199867020089)

[62] Mukherjee S, Maitra SK. 2015 Gut melatonin in vertebrates: chronobiology and physiology. Front. Endocrinol. (Lausanne) **6**, 112. (10.3389/fendo.2015.00112) 26257705 PMC4510419

[63] Zhang Y et al. 2023 Reducing light exposure enhances the circadian rhythm of the biological clock through interactions with the gut microbiota. Sci. Total Environ. **858**, 160041. (10.1016/j.scitotenv.2022.160041) 36356756

[64] Silverin B, Gwinner E, Van't Hof TJ, Schwabl I, Fusani L, Hau M, Helm B. 2009 Persistent diel melatonin rhythmicity during the Arctic summer in free-living willow warblers. Horm. Behav. **56**, 163-168. (10.1016/j.yhbeh.2009.04.002) 19374903

[65] Wikelski M, Tarlow EM, Eising CM, Groothuis TGG, Gwinner E. 2006 Do night-active birds lack daily melatonin rhythms? A case study comparing a diurnal and a nocturnal-foraging gull species. J. Ornithol. **147**, 107-111. (10.1007/s10336-005-0018-4)

[66] Dominoni D, Goymann W, Helm B, Partecke J. 2013 Urban-like night illumination reduces melatonin release in European blackbirds (*Turdus merula*): implications of city life for biological time-keeping of songbirds. Front. Zool. **10**, 60. (10.1186/1742-9994-10-60) 24090446 PMC3850952

[67] Gwinner E, Schwabl-Benzinger I, Schwabl H, Dittami J. 1993 Twenty-four hour melatonin profiles in a nocturnally migrating bird during and between migratory seasons. Gen. Comp. Endocrinol. **90**, 119-124. (10.1006/gcen.1993.1066) 8504916

[68] Tarlow EM, Hau M, Anderson DJ, Wikelski M. 2003 Diel changes in plasma melatonin and corticosterone concentrations in tropical Nazca boobies (*Sula granti*) in relation to moon phase and age. Gen. Comp. Endocrinol. **133**, 297-304. (10.1016/S0016-6480(03)00192-8) 12957473

[69] Helm B, Gwinner E, Koolhaas A, Battley P, Schwabl I, Dekinga A, Piersma T. 2012 Avian migration: temporal multitasking and a case study of melatonin cycles in waders. In Progress in brain research (ed. A Kalsbeek), pp. 457-479. Amsterdam, The Netherlands: Elsevier. 10.1016/B978-0-444-59427-3.00026-5 22877681

[70] Gwinner E, Brandstätter R. 2001 Complex bird clocks. Phil. Trans. R. Soc. B **356**, 1801-1810. (10.1098/rstb.2001.0959) 11710987 PMC1088556

[71] Van't Hof TJ, Gwinner E, Wagner H. 1998 A highly rudimentary circadian melatonin profile in a nocturnal bird, the barn owl (*Tyto alba*). Naturwissenschaften **85**, 402-404. (10.1007/s001140050523)

[72] Ashley NT, Ubuka T, Schwabl I, Goymann W, Salli BM, Bentley GE, Buck CL. 2014 Revealing a circadian clock in captive arctic-breeding songbirds, lapland longspurs (*Calcarius lapponicus*), under constant illumination. J. Biol. Rhythms **29**, 456-469. (10.1177/0748730414552323) 25326246

[73] Gwinner E, Hau M, Heigl S. 1997 Melatonin: generation and modulation of avian circadian rhythms. Brain Res. Bull. **44**, 439-444. (10.1016/S0361-9230(97)00224-4) 9370209

[74] Zeman M, Buyse J, Herichovã I, Decuypere E. 2001 Melatonin decreases heat production in female broiler chickens. Acta Vet. Brno **70**, 15-18. (10.2754/avb200170010015)

[75] Nakahara K, Kawano T, Shiota K, Murakami N. 2003 Effects of microinjection of melatonin into various brain regions of Japanese quail on locomotor activity and body temperature. Neurosci. Lett. **345**, 117-120. (10.1016/S0304-3940(03)00514-7) 12821185

[76] Oshima I, Yamada H, Goto M, Sato K, Ebihara S. 1989 Pineal and retinal melatonin is involved in the control of circadian locomotor activity and body temperature rhythms in the pigeon. J. Comp. Physiol. A **166**, 217-226. (10.1007/BF00193466)

[77] Murakami N, Kawano T, Nakahara K, Nasu T, Shiota K. 2001 Effect of melatonin on circadian rhythm, locomotor activity and body temperature in the intact house sparrow, Japanese quail and owl. Brain Res. **889**, 220-224. (10.1016/S0006-8993(00)03205-4) 11166707

[78] Kharwar RK, Haldar C. 2012 Daily variation in antioxidant enzymes and lipid peroxidation in lungs of a tropical bird *Perdicula asiatica*: role of melatonin and nuclear receptor ROR*α*. Comp. Biochem. Physiol. A **162**, 296-302. (10.1016/j.cbpa.2012.01.021) 22349119

[79] Siopes TD, Underwood HA. 2008 Diurnal variation in the cellular and humoral immune responses of Japanese quail: role of melatonin. Gen. Comp. Endocrinol. **158**, 245-249. (10.1016/j.ygcen.2008.07.008) 18703065

[80] Berthold P. 2001 Bird migration: a general survey. Oxford, UK: Oxford University Press.

[81] Fusani L, Gwinner E. 2005 Melatonin and nocturnal migration. Ann. N Y Acad. Sci. **1046**, 264-270. (10.1196/annals.1343.024) 16055859

[82] Pohl H. 2000 Circadian control of migratory restlessness and the effects of exogenous melatonin in the brambling (*Fringilla montfiringilla*). Chronobiol. Int. **17**, 471-488. (10.1081/CBI-100101058) 10908124

[83] Fusani L, Cardinale M, Schwabl I, Goymann W. 2011 Food availability but not melatonin affects nocturnal restlessness in a wild migrating passerine. Horm. Behav. **59**, 187-192. (10.1016/j.yhbeh.2010.11.013) 21110977

[84] Fusani L, Coccon F, Rojas Mora A, Goymann W. 2013 Melatonin reduces migratory restlessness in Sylvia warblers during autumnal migration. Front. Zool. **10**, 79. (10.1186/1742-9994-10-79) 24369961 PMC3879198

[85] Goldman BD. 2001 Mammalian photoperiodic system: formal properties and neuroendocrine mechanisms of photoperiodic time measurement. J. Biol. Rhythms **16**, 283-301. (10.1177/074873001129001980) 11506375

[86] Gwinner E, Dittami J. 1982 Pineal influences on circannual cycles in European starlings: effects through the circadian system?. In Vertebrate circadian systems: structure and physiology (eds J Aschoff, S Daan, G Groos), pp. 276-284. Berlin, Germany: Springer.

[87] Dawson A, King VM, Bentley GE, Ball GF. 2001 Photoperiodic control of seasonality in birds. J. Biol. Rhythms **16**, 365-380. (10.1177/074873001129002079) 11506381

[88] Ohta M, Kadota C, Konishi H. 1989 A role of melatonin in the initial stage of photoperiodism in the Japanese quail. Biol. Reprod. **40**, 935-941. (10.1095/biolreprod40.5.935) 2765617

[89] Juss TS, Meddle SL, Servant RS, King VM. 1993 Melatonin and photoperiodic time measurement in Japanese quail (*Coturnix coturnix japonica*). Proc. R. Soc. Lond. B **254**, 21-28. (10.1098/rspb.1993.0121) 8265672

[90] McGuire NL, Kangas K, Bentley GE. 2011 Effects of melatonin on peripheral reproductive function: regulation of testicular GnIH and testosterone. Endocrinology **152**, 3461-3470. (10.1210/en.2011-1053) 21771888

[91] Ubuka T, Bentley GE, Ukena K, Wingfield JC, Tsutsui K. 2005 Melatonin induces the expression of gonadotropin-inhibitory hormone in the avian brain. Proc. Natl Acad. Sci. USA **102**, 3052-3057. (10.1073/pnas.0403840102) 15708982 PMC549437

[92] Bentley GE, Van't Hof TJ, Ball GF. 1999 Seasonal neuroplasticity in the songbird telencephalon: a role for melatonin. Proc. Natl Acad. Sci. USA **96**, 4674-4679. (10.1073/pnas.96.8.4674) 10200321 PMC16391

[93] Jones C, Helfer G, Brandstätter R. 2012 Melatonin receptor expression in the zebra finch brain and peripheral tissues. Chronobiol. Int. **29**, 189-202. (10.3109/07420528.2011.642912) 22324557

[94] Greives TJ, Kingma SA, Beltrami G, Hau M. 2012 Melatonin delays clutch initiation in a wild songbird. Biol. Lett. **8**, 330-332. (10.1098/rsbl.2011.1100) 22171024 PMC3367749

[95] Greives TJ et al. 2015 Costs of sleeping in: circadian rhythms influence cuckoldry risk in a songbird. Funct. Ecol. **29**, 1300-1307. (10.1111/1365-2435.12440)

[96] Menzel A, Sparks TH, Estrella N, Roy DB. 2006 Altered geographic and temporal variability in phenology in response to climate change. Global Ecol. Biogeogr. **15**, 498-504. (10.1111/j.1466-822X.2006.00247.x)

[97] Regan CE, Sheldon BC. 2023 Phenotypic plasticity increases exposure to extreme climatic events that reduce individual fitness. Glob. Change Biol. **29**, 2968-2980. (10.1111/gcb.16663) PMC10947444 36867108

[98] Shipley JR, Twining CW, Taff CC, Vitousek MN, Flack A, Winkler DW. 2020 Birds advancing lay dates with warming springs face greater risk of chick mortality. Proc. Natl Acad. Sci. USA **117**, 25 590-25 594. (10.1073/pnas.2009864117) PMC7568286 32989166

[99] Sumova A, Sladek M, Polidarova L, Novakova M, Houdek P. 2012 Circadian system from conception till adulthood. Prog. Brain Res. **199**, 83-103. (10.1016/B978-0-444-59427-3.00005-8) 22877660

[100] Lamosová D, Zeman M, Mackova M, Gwinner E. 1995 Development of rhythmic melatonin synthesis in cultured pineal glands and pineal cells isolated from chick embryo. Experientia **51**, 970-975. (10.1007/BF01921750) 7556580

[101] Hanuszewska M, Prusik M, Lewczuk B. 2019 Embryonic ontogeny of 5-hydroxyindoles and 5-methoxyindoles synthesis pathways in the goose pineal organ. Int. J. Mol. Sci. **20**, 3948. (10.3390/ijms20163948) 31416134 PMC6719024

[102] Zeman M, Illnerová H. 1990 Ontogeny of N-acetyltransferase activity rhythm in pineal gland of chick embryo. Comp. Biochem. Physiol. A **97**, 175-178. (10.1016/0300-9629(90)90166-P) 1982932

[103] Zeman M, Gwinner E, Herichová I, Lamošová D, Košt'ál L. 1999 Perinatal development of circadian melatonin production in domestic chicks. J. Pineal Res. **26**, 28-34. (10.1111/j.1600-079X.1999.tb00563.x) 10102757

[104] Okabayashi N, Yasuo S, Watanabe M, Namikawa T, Ebihara S, Yoshimura T. 2003 Ontogeny of circadian clock gene expression in the pineal and the suprachiasmatic nucleus of chick embryo. Brain Res. **990**, 231-234. (10.1016/S0006-8993(03)03531-5) 14568350

[105] Herichová I, Monosíková J, Zeman M. 2008 Ontogeny of melatonin, Per2 and E4bp4 light responsiveness in the chicken embryonic pineal gland. Comp. Biochem. Physiol. A **149**, 44-50. (10.1016/j.cbpa.2007.10.006) 17996471

[106] Zeman M, Gwinner E. 1993 Ontogeny of the rhythmic melatonin production in a precocial and an altricial bird, the Japanese quail and the European starling. J. Comp. Physiol. A **172**, 333-338. (10.1007/BF00216615)

[107] Van't Hof TJ, Gwinner E. 1996 Development of post-hatching melatonin rhythm in zebra finches (*Poephila guttata*). Experientia **52**, 249-252. (10.1007/BF01920717) 8631396

[108] Zeman M, Herichová I. 2011 Circadian melatonin production develops faster in birds than in mammals. Gen. Comp. Endocrinol. **172**, 23-30. (10.1016/j.ygcen.2010.12.022) 21199656

[109] Höchel J, Nichelmann M. 2001 Ontogeny of heart rate responses to exogenous melatonin in Muscovy duck and chicken embryos. Life Sci. **69**, 2295-2309. (10.1016/S0024-3205(01)01302-9) 11669472

[110] Haraguchi S, Tsutsui K. 2020 Pineal neurosteroids: biosynthesis and physiological functions. Front. Endocrinol. **11**, 549. (10.3389/fendo.2020.00549) PMC7431617 32849313

[111] Tsutsui K, Inoue K, Miyabara H, Suzuki S, Ogura Y, Haraguchi S. 2008 7Alpha-hydroxypregnenolone mediates melatonin action underlying diurnal locomotor rhythms. J. Neurosci. **28**, 2158-2167. (10.1523/JNEUROSCI.3562-07.2008) 18305249 PMC6671851

[112] Haim A, Zubidat AE. 2015 Artificial light at night: melatonin as a mediator between the environment and epigenome. Phil. Trans. R. Soc. B **370**, 20140121. (10.1098/rstb.2014.0121) 25780234 PMC4375362

[113] Formanek L, Richard-Yris MA, Houdelier C, Lumineau S. 2009 Epigenetic maternal effects on endogenous rhythms in precocial birds. Chronobiol. Int. **26**, 396-414. (10.1080/07420520902892433) 19360486

[114] Gwinner E, Zeman M, Klaassen M. 1997 Synchronization by low-amplitude light–dark cycles of 24-hour pineal and plasma melatonin rhythms of hatchling European starlings (*Sturnus vulgaris*). J. Pineal Res. **23**, 176-181. (10.1111/j.1600-079X.1997.tb00352.x) 9462849

[115] Griffith SC, Mainwaring MC, Sorato E, Beckmann C. 2016 High atmospheric temperatures and ‘ambient incubation’ drive embryonic development and lead to earlier hatching in a passerine bird. R. Soc. Open Sci. **3**, 150371. (10.1098/rsos.150371) 26998315 PMC4785966

[116] Drozdova A, Okuliarova M, Zeman M. 2019 The effect of different wavelengths of light during incubation on the development of rhythmic pineal melatonin biosynthesis in chick embryos. Animal **13**, 1635-1640. (10.1017/S1751731118003695) 30614433

[117] Zeman M, Lamosova D, Herichová I, Gwinner E, Pavlik P. 2004 Entrainment of rhythmic melatonin production by light and temperature in the chick embryo. Avian Poultry Biol. Rev. **15**, 197-204. (10.3184/147020604783638155)

[118] Rowan W. 1937 Effects of traffic disturbance and light illumination on London starlings. Nature **139**, 668-669. (10.1038/139668a0)

[119] Calisi RM, Bentley GE. 2009 Lab and field experiments: are they the same animal? Horm. Behav. **56**, 1-10. (10.1016/j.yhbeh.2009.02.010) 19281813

[120] Zolotareva A, Utvenko G, Romanova N, Pakhomov A, Chernetsov N. 2021 Ontogeny of the star compass in birds: pied flycatchers (*Ficedula hypoleuca*) can establish the star compass in spring. J. Exp. Biol. **224**, jeb237875. (10.1242/jeb.237875) 33436368

[121] Van Doren BM, Horton KG, Dokter AM, Klinck H, Elbin SB, Farnsworth A. 2017 High-intensity urban light installation dramatically alters nocturnal bird migration. Proc. Natl Acad. Sci. USA **114**, 11 175-11 180. (10.1073/pnas.1708574114) 28973942 PMC5651764

[122] Korner P, von Maravic I, Haupt H. 2022 Birds and the ‘Post Tower’ in Bonn: a case study of light pollution. J. Ornithol. **163**, 827-841. (10.1007/s10336-022-01985-2)

[123] Jägerbrand AK, Spoelstra K. 2023 Effects of anthropogenic light on species and ecosystems. Science **380**, 1125-1130. (10.1126/science.adg3173) 37319223

[124] Canário F, Leitão AH, Tomé R. 2012 Predation attempts by short-eared and long-eared owls on migrating songbirds attracted to artificial lights. J. Raptor Res. **46**, 232-234. (10.3356/JRR-11-15.1)

[125] Mishra I, Knerr RM, Stewart AA, Payette WI, Richter MM, Ashley NT. 2019 Light at night disrupts diel patterns of cytokine gene expression and endocrine profiles in zebra finch (*Taeniopygia guttata*). Sci. Rep. **9**, 15833. (10.1038/s41598-019-51791-9) 31676761 PMC6825233

[126] Partecke J, Vańt Hof TJ, Gwinner E. 2004 Differences in the timing of reproduction between urban and forest European blackbirds (*Turdus merula*)—result of phenotypic flexibility or genetic differences? Proc. R. Soc. B **271**, 1995-2001. (10.1098/rspb.2004.2821) PMC1691820 15451688

[127] Dominoni D, Quetting M, Partecke J. 2013 Artificial light at night advances avian reproductive physiology. Proc. Biol. Sci. **280**, 20123017. 23407836 10.1098/rspb.2012.3017 PMC3574380

[128] Ziegler A-K, Watson H, Hegemann A, Meitern R, Canoine V, Nilsson JÅ, Isaksson C. 2021 Exposure to artificial light at night alters innate immune response in wild great tit nestlings. J. Exp. Biol. **224**, jeb239350. (10.1242/jeb.239350) 33771912 PMC8180251

[129] Kernbach ME, Cassone VM, Unnasch TR, Martin LB. 2020 Broad-spectrum light pollution suppresses melatonin and increases West Nile virus-induced mortality in house sparrows (*Passer domesticus*). The Condor **122**, duaa018. (10.1093/condor/duaa018)

[130] Becker DJ, Singh D, Pan Q, Montoure JD, Talbott KM, Wanamaker SM, Ketterson ED. 2020 Artificial light at night amplifies seasonal relapse of haemosporidian parasites in a widespread songbird. Proc. R. Soc. B **287**, 20201831. (10.1098/rspb.2020.1831) PMC7542808 32962545

[131] Martinez-Bakker M, Helm B. 2015 The influence of biological rhythms on host–parasite interactions. Trends Ecol. Evol. **30**, 314-326. (10.1016/j.tree.2015.03.012) 25907430

[132] Kennaway DJ. 2020 Measuring melatonin by immunoassay. J. Pineal Res. **69**, e12657. (10.1111/jpi.12657) 32281677

[133] de Jong M, Jeninga L, Ouyang JQ, Van Oers K, Spoelstra K, Visser ME. 2016 Dose-dependent responses of avian daily rhythms to artificial light at night. Physiol. Behav. **155**, 172-179. (10.1016/j.physbeh.2015.12.012) 26703233

[134] Moaraf S, Vistoropsky Y, Pozner T, Heiblum R, Okuliarová M, Zeman M, Barnea A. 2019 Artificial light at night affects brain plasticity and melatonin in birds. Neurosci. Lett. **716**, 134639. (10.1016/j.neulet.2019.134639) 31760086

[135] Kumar J, Malik S, Bhardwaj SK, Rani S. 2021 Impact of light at night is phase dependent: a study on migratory redheaded bunting (*Emberiza bruniceps*). Front. Ecol. Evol. **9**, 751072. (10.3389/fevo.2021.751072)

[136] Dzirbíková Z, Stebelová K, Kováčová K, Okuliarová M, Olexová L, Zeman M. 2022 Artificial dim light at night during pregnancy can affect hormonal and metabolic rhythms in rat offspring. Int. J. Mol. Sci. **23**, 14544. (10.3390/ijms232314544) 36498872 PMC9740453

[137] Mendez N et al. 2016 Gestational chronodisruption impairs circadian physiology in rat male offspring, increasing the risk of chronic disease. Endocrinology **157**, 4654-4668. (10.1210/en.2016-1282) 27802074

[138] Helm B, Greives T, Zeman M. 2023 Endocrine–circadian interactions in birds: implications when nights are no longer dark. *Figshare*. (10.6084/m9.figshare.c.7007959)38310930

